# Characterization of the volatile components in green tea by IRAE-HS-SPME/GC-MS combined with multivariate analysis

**DOI:** 10.1371/journal.pone.0193393

**Published:** 2018-03-01

**Authors:** Yan-Qin Yang, Hong-Xu Yin, Hai-Bo Yuan, Yong-Wen Jiang, Chun-Wang Dong, Yu-Liang Deng

**Affiliations:** Key Laboratory of Tea Biology and Resources Utilization, Ministry of Agriculture, Tea Research Institute, Chinese Academy of Agricultural Sciences, Hangzhou, Zhejiang, China; Agricultural University of Athens, GREECE

## Abstract

In the present work, a novel infrared-assisted extraction coupled to headspace solid-phase microextraction (IRAE-HS-SPME) followed by gas chromatography-mass spectrometry (GC-MS) was developed for rapid determination of the volatile components in green tea. The extraction parameters such as fiber type, sample amount, infrared power, extraction time, and infrared lamp distance were optimized by orthogonal experimental design. Under optimum conditions, a total of 82 volatile compounds in 21 green tea samples from different geographical origins were identified. Compared with classical water-bath heating, the proposed technique has remarkable advantages of considerably reducing the analytical time and high efficiency. In addition, an effective classification of green teas based on their volatile profiles was achieved by partial least square-discriminant analysis (PLS-DA) and hierarchical clustering analysis (HCA). Furthermore, the application of a dual criterion based on the variable importance in the projection (VIP) values of the PLS-DA models and on the category from one-way univariate analysis (ANOVA) allowed the identification of 12 potential volatile markers, which were considered to make the most important contribution to the discrimination of the samples. The results suggest that IRAE-HS-SPME/GC-MS technique combined with multivariate analysis offers a valuable tool to assess geographical traceability of different tea varieties.

## Introduction

Tea is one of the most popular beverages in the world owing to its attractive aroma, taste, and health benefits [1, 2]. According to the way of processing, teas are usually classified into three big groups based on their fermentation degrees: non-fermented (green and white), semi-fermented (oolong) and fully fermented (black tea including pu-erh tea) [3]. Of these, green tea has gained more popularity because of its pleasant flavor, mainly in Asian countries especially in Japan and China [4]. The manufacturing process of green tea basically involves four steps, including withering, pan firing, rolling, and drying. According to the heating process to inactivate the endogenous enzymes in the leaves, green tea is mainly divided into two types, i.e., pan-firing and steaming [5]. In China, there are numerous green tea cultivars classified by the subtle differences in the producing area, varieties of tea, and/or the manufacturing process, which characterizes the colour, appearance and flavor of the final product. For this reason, most tea products are marketed with the indication of the production region for product authentication and valorization [6].

Aroma is one of the important indicators for the quality evaluation of teas [7]. The commonly used analytical techniques for aroma analysis in the tea industry involve gas chromatography coupled to mass spectrometry (GC-MS), gas chromatography-olfactometry (GC-O), and electronic nose (E-nose) [8]. GC-MS is one of the most widely used techniques for the analysis and profiling of aroma compounds owing to its excellent performance in separating, identifying, and quantifying individual volatile compounds from complex systems [9]. In recent years, GC-MS combined with multivariate analysis such as principal component analysis (PCA), hierarchical cluster analysis (HCA), similarity analysis (SA), and partial least squares-discrimination analysis (PLS-DA) has been widely implemented in metabolomics analysis [10], chemical classification [11], quality control [12], and chromatographic profile aligning [13, 14].

The volatile components represent only about 0.01% of the total dry weight of tea, but they play an important role in determining the flavor of the products due to their low threshold value and high odor sensibility [15]. Up to date, many extraction methods such as steam distillation, soxhlet extraction, simultaneous distillation extraction (SDE) and direct organic solvent extraction have been used to characterize the volatile components in teas [7]. These techniques provide reliable sample profiles but have some drawbacks such as time-consuming, low extracting efficiency, toxic solvent, and even the analyte degradation produced by solvents, and oxidation (oxygen effect) and temperature triggered reactions [16]. Therefore, it is of great importance to develop a simple, rapid, solvent-free, and low-cost extraction method to act as an alternative to conventional techniques for the tea industry. Headspace solid-phase microextraction (HS-SPME) is a very promising pre-treatment method, for its remarkable analytical characteristics, including good linearity, reproducibility, and low limits of detection. It integrates sampling, pre-concentration and sample introduction in a single and uninterrupted step. In recent years, HS-SPME has been successfully applied to characterize the volatile profiles in medicines [17], food [18, 19], fruit [20, 21] and wines [22].

Microwave assisted extraction (MAE) is a relatively new extraction technique and has been widely used in food and natural product analysis, due to its special heating mechanism and good performance. It consists of electric field and magnetic field which oscillates perpendicularly to each other in frequency ranged from 0.3 to 300 GHz. However, the microwave radiation should be handled carefully because the leaked microwaves are dangerous to human health [23, 24].

As an important form of electromagnetic wave, infrared (IR) radiation has wavelengths ranging from 750 nm to 1 mm, lying between visible radiation and microwaves [25]. By matching the wavelength of the IR irradiation to the absorption characteristics of the materials, the high efficiency of the IR irradiation is achieved [26]. With the advantages of safe and simple operation, high permeability, low energy consumption as well as rapid heating, IR irradiation is widely applied in industrial heating, communications, military, spectroscopic analysis, and health care, nowadays [27]. IR irradiation could improve the extraction efficiency of analytes compared with conventional techniques. Recently, Duan and colleagues successfully employed IR radiation to enhance the efficiency of extracts from Lycium barbarum Linn, and the extraction time for the investigated herbal was strikingly reduced to 6 min compared with 3 h for conventional hot solvent extraction [28]. Therefore, employing IR radiation to improve the efficiency of HS-SPME technique is feasible and promising. To the best of the authors' knowledge, no references have been reported so far concerning the volatile profile of green tea by coupling infrared-assisted extraction with HS-SPME technique.

In the present work, infrared-assisted extraction coupled to headspace solid-phase microextraction (IRAE-HS-SPME) has been developed for the rapid analysis of the volatile constituents in green tea based on GC-MS. To our best knowledge, this is the first report of exploring IRAE-HS-SPME on the aroma composition in green tea. The extraction parameters such as fiber type, sample amount, IR power, extraction time, and IR lamp distance were investigated by orthogonal experimental design to acquire the optimal analysis conditions. A comparison between IR radiation and conventional water-bath heating was conducted. Multivariate statistical techniques such as PLS-DA, HCA were implemented to understand the potential characteristics according to the geographical origins of the samples. The application of a dual criterion based on variable importance in projection (VIP) scores and the results of one-way univariate analysis (ANOVA), allowed the identification of potential volatile markers from different geographical origins.

## Materials and methods

### Materials and reagents

In total, 21 commercially available roasted green tea samples were purchased from the local market. The detailed name and sources were listed in S1 Table. All tea samples were stored in a refrigerator at a temperature below -20 °C until analyses were performed.

The SPME manual holder and the fibers of polyacrylate (PA, 85 μm), polydimethylsiloxane-divinylbenzene (PDMS–DVB, 65μm), and carboxen-polydimethylsiloxane (CAR-PDMS, 75 μm) were purchased from Supelco, USA. The n-alkane mixtures, consisting of C7–C40 straight-chain alkanes, were supplied by Supelco, USA.

### Sample preparation

#### Infrared-assisted extraction coupled to headspace solid-phase microextraction method

**Fig 1:**
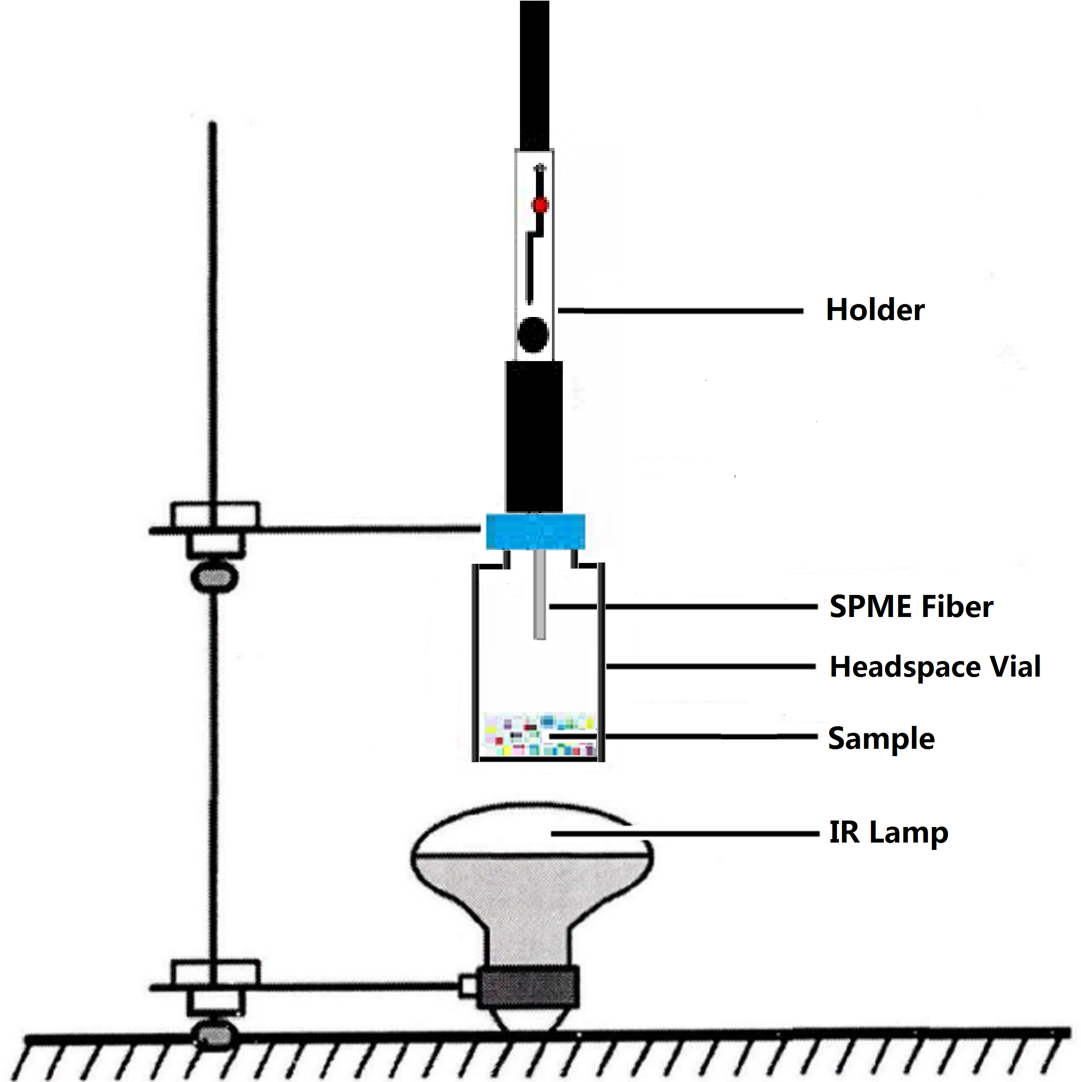
The apparatus of IRAE- HS-SPME system.

The infrared-assisted extraction system is shown in Fig 1. The IR lamp (Qiyi Lighting Company, Zhejiang, China) was laid below the headspace vial as the heat resource to extract the volatile components of green tea.

Prior to use, the fibers were conditioned by inserting them into the GC injector port according to the manufacture instructions: 0.5 h at 280 °C for PA, 0.5 h at 250 °C for PDMS-DVB, and 1 h at 300 °C for CAR-PDMS, respectively. Before sampling, each fiber was reconditioned for 5 min in the GC injector port at 250 °C to eliminate the possible remains on the fiber coating.

The sample (0.5 g) was quickly introduced into 20-mL headspace vial and the vial was hermetically closed by a teflon/silicone septum. The needle coated with different fibers was inserted into the vials by manually penetrating the septum and the fibers were exposed to the headspace above the samples. After a period of extracting time at setting infrared power, the needle was removed from the headspace vial and directly inserted into the injection port of GC-MS. The effects of different fiber types (PA, PDMS-DVB and CAR-PDMS), sample amount (0.1, 0.3, and 0.5 g), IR power (100, 175, and 250 W), extraction time (10, 15, and 20 min), the distance between the IR lamp and the headspace vial (6, 8, and 10 cm) were investigated.

#### Water-bath heating headspace solid-phase microextraction method

The sample (0.5 g) was quickly introduced into 20-mL headspace vial and the vial was hermetically closed by home-made septum and immersed in a water bath. The needle coated with CAR-PDMS fiber was inserted into the vial by manually piercing the septum and the fiber was exposed to the headspace above the samples. After 20 min of extracting time at equality temperature of 90 °C, the needle was removed from the headspace vial and directly inserted into the injection port of GC-MS. The analytes were thermally desorbed in the splitless mode at 250 °C for 5 min.

### Gas chromatography-mass spectrometry

GC-MS was performed with an Agilent Technologies 7890A GC system coupled to an Agilent Technologies 7000C Triple Quadrupole mass spectrometer. Samples were analyzed on a HP-5ms (30 m ×I.D. 0.25 mm × film thickness 0.25 μm). Analytical conditions were as follows: The oven temperature was held at 40 °C for 0 min, and raised to 190 °C for 2 min at 3 °C/min, then increased to 290 °C at 10 °C/min, maintaining for 3 min; Splitless mode was conducted; Helium (99.999% purity) was used as the carrier gas at a rate of 1 mL/min.

The mass spectrometer was operated in an electron-impact (EI) mode. The scan range was 50–450 m/z. The scan rate was 0.2s/scan. The temperatures at ionization source and interface were 230 and 280 °C, respectively.

### Qualitative analysis

The qualitative analysis of the volatile components was processed by Agilent MassHunter Workstation Software Unknowns Analysis with the ability of peak picking, peak deconvolution and mass spectra comparison. The compounds were identified by comparing the obtained spectra with those of reference compounds from the National Institute of Standards and Technology (NIST14) and by the Kovàts retention indices calculated for each peak with reference to the n-alkane standards (C7–C40) running under the same conditions. Peaks were assigned when the similarity was above 80%. Any known artificial peaks were excluded from the data set. The relative percentages of various components in the samples were obtained by peak area normalization.

### Statistical methods

Significant differences among green tea samples were determined by one-way ANOVA using SPSS 18.0 (SPSS Inc., USA). The difference between two related results was considered to be statistically significant with values of p<0.05. HCA was also performed using the SPSS 18.0 (SPSS Inc., USA). PLS-DA was conducted by SIMCA-P software with the version 11 (UMETRICS, Sweden). All variables were scaled with unit variance (UV) prior to PLS-DA.

#### Partial least squares-discrimination analysis

PLS-DA was conducted to develop models to find a two dimensional plane (discriminating plane) in which the tea samples (projected observations) on the PLS components were well separated according to their volatile compounds. As for the PLS weight plot, composition variables of weight plot can reveal the variables (specific volatile compounds) contributing to the separation.

#### Hierarchical clustering analysis

HCA is an unsupervised chemometric technique that reveals the natural groupings existing between samples characterised by the values of a set of measured variables. The results achieved were described by a dendrogram representing in a tree structure. In the present work, the similarities between samples were calculated on the basis of the Squared Euclidean distance, whereas Ward’s method was used as a linkage procedure to establish the clusters.

## Results and discussion

### Optimization of the infrared-assisted extraction coupled to headspace solid-phase microextraction parameters

The extraction process of IRAE-HS-SPME was affected by multiple parameters. In order to acquire the maximum extraction efficiency, a number of experiments under different conditions were performed. The effects of fiber type, sample amount, infrared power, extraction time, and the IR lamp distance on the extraction efficiency were investigated by orthogonal experimental design.

#### Headspace solid-phase microextraction fiber

**Fig 2:**
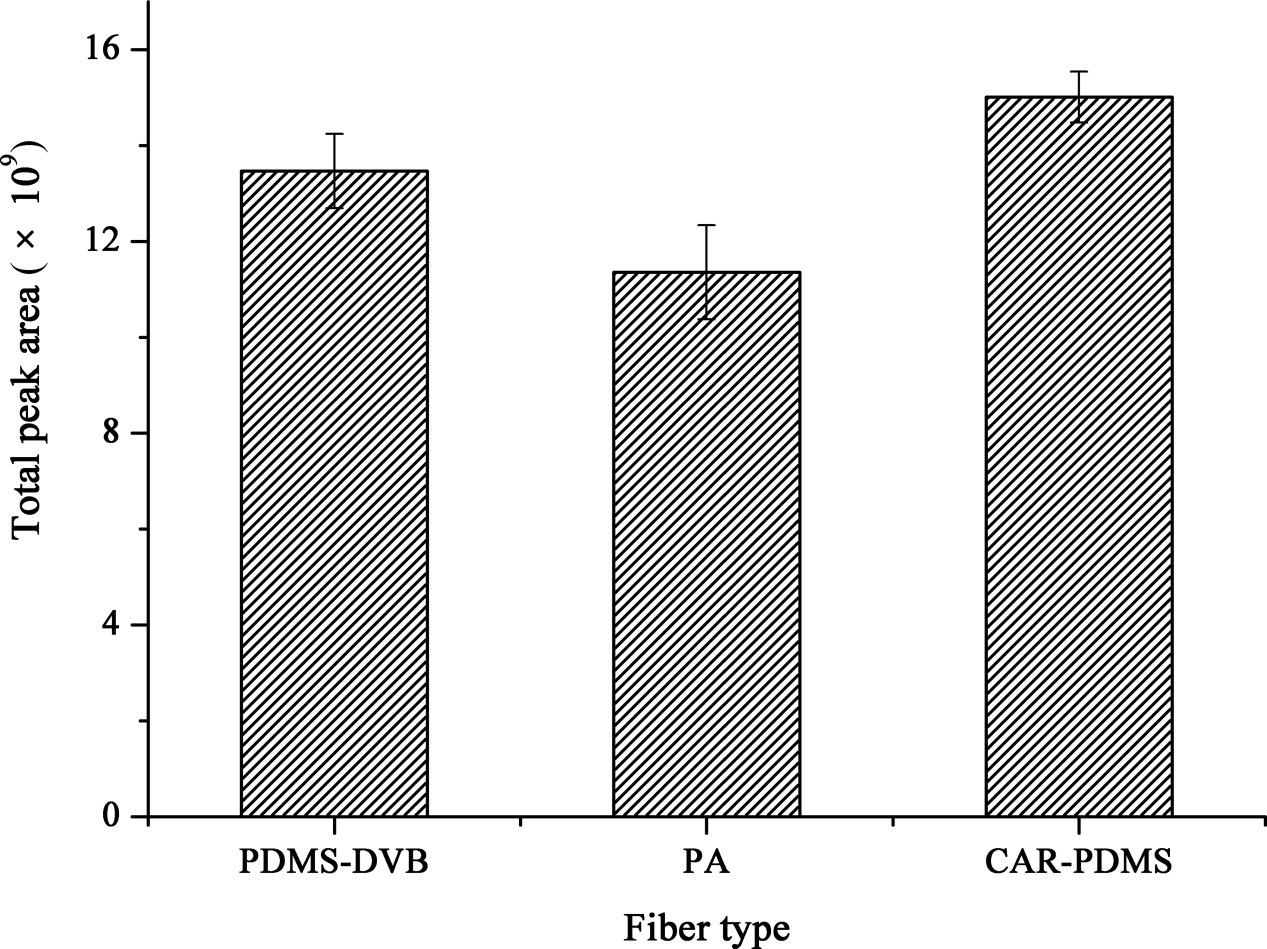
The total peak areas and peak number obtained by different fiber coatings.

The fiber coating is the “heart” of the extraction. In order to evaluate the selectivity of different fiber coatings, three types of fibers (CAR-PDMS, PDMS-DVB, and PA) with different polarity and inner structure were tested for the extraction efficiency of the volatile compounds in green tea. The total peak areas using PA and PDMS-DVB fibers were 76.66% and 89.72% of that using CAR-PDMS respectively (see Fig 2), indicating that the CAR-PDMS fiber was the most efficient and had the strongest retention ability for the volatile compounds in green tea. In addition, CAR-PDMS fiber showed the best repeatability. Therefore, the CAR-PDMS fiber was regarded as the optimal fiber and was employed in the following study.

#### Orthogonal experimental design

In this work, a three-level orthogonal array design with an L9 (3^4^) matrix was conducted to optimize the sample amount (factor A, from 0.1 to 0.5 g), extraction time (factor B, from 10 to 20 min), IR lamp power (factor C, from 100 to 275 W), and IR lamp distance (factor D, from 6 to 10 cm) (see Table 1). Compared to the traditional optimization process that arrays one parameter each time, the orthogonal experiment design is regarded as the modern approach to characterize and optimize system performance in many research areas. The orthogonal experiment design method can be used to select representative points from the full factorial experiments which are distributed uniformly within the test range and thus can adequately represent the overall situation [29, 30].

**Table 1:** The results and analysis of orthogonal design L9 (3^4^).

No.	Factor A- Sample amount (g)	Factor B- Extraction time (min)	Factor C-IR lamp power (W)	Factor D-IR lamp distance (cm)	Sum of peak area × 10^9^
1	0.10	10	100	6	2.54
2	0.10	15	175	8	7.31
3	0.10	20	250	10	5.26
4	0.30	10	175	10	4.78
5	0.30	15	250	6	11.25
6	0.30	20	100	8	4.59
7	0.50	10	250	8	5.41
8	0.50	15	100	10	3.40
9	0.50	20	175	6	15.01
K1	5.037	4.243	3.517	9.600	
K2	6.873	7.327	9.033	5.770	
K3	7.947	8.287	7.307	4.487	
k1	1.679	1.414	1.172	3.200	
k2	2.291	2.442	3.011	1.923	
k3	2.649	2.762	2.436	1.496	
R	0.970	1.348	1.839	1.704	

In this study, the orthogonal experiment design was used to evaluate the inner relationship and the influence sequence among factors, which might significantly affect the extraction efficiency. The Ki data was obtained by averaging the total peak area in the same level of each factor. The ki data was obtained by Ki data divided by the level number. The R value for each factor was calculated by finding the difference between maximum and minimum k value. The larger the R value for a factor, the stronger is the influence of the test factor on the result. The results suggest that the order of influence for extraction efficiency is RC > RD > RB > RA. In other words, the IR lamp power has the most significant impact on the detected amounts of volatile compounds in green tea followed by IR lamp distance, extraction time, and sample amount.

The variation trend of ki can be used for determining the optimal level. From the results in Table 1, we conclude that k3 > k2 > k1 for Factor A, k3 > k2 > k1 for Factor B, k2 > k3 > k1 for Factor C and k3 < k2 < k1 for Factor D. Therefore, the optimal combination of factor levels will be A3B3C2D1. In other words, a higher extraction efficiency will be achieved for sample amount of 0.5 g, extraction time of 20 min, IR power of 175 W and IR lamp distance of 6 cm. The standard statistical technique of ANOVA was used to estimate the relative significance of each parameter in terms of percentage contribution to the overall response. By comparing the F-value of different factors, it is obvious that the order of factors from large to small is CDBA (see Table 2). The order of factors determined in the ANOVA analysis is the same as for R value, which verifies the previously calculated results.

**Table 2:** The results of variance analysis.

Error sources	SS	*f*	*S*	*F*	*Significance*
Factor A-**Sample amount**	12.993	2	6.496	19.508	<0.01
Factor B-**Extraction time**	26.777	2	13.388	40.204	<0.01
Factor C-**IR lamp power**	47.779	2	23.890	71.742	<0.01
Factor D-**IR lamp distance**	42.462	2	21.231	63.757	<0.01
**Error**	6.000	18	0.333		

*F*_0.01_(2,18) = 6.01; **SS**: Sum of squares of deviations; ***f***: Degrees of freedom; ***S*:** Mean squares; ***F*:** F variance ratio; ***P*:** Significance

Overall, the optimized extraction conditions were as follows: CAR-PDMS fiber, sample amount of 0.5 g, extraction time of 20 min, IR power of 175 W and IR lamp distance of 6 cm.

### Analysis of the volatile compounds in green teas from different geographical origins

**Fig 3:**
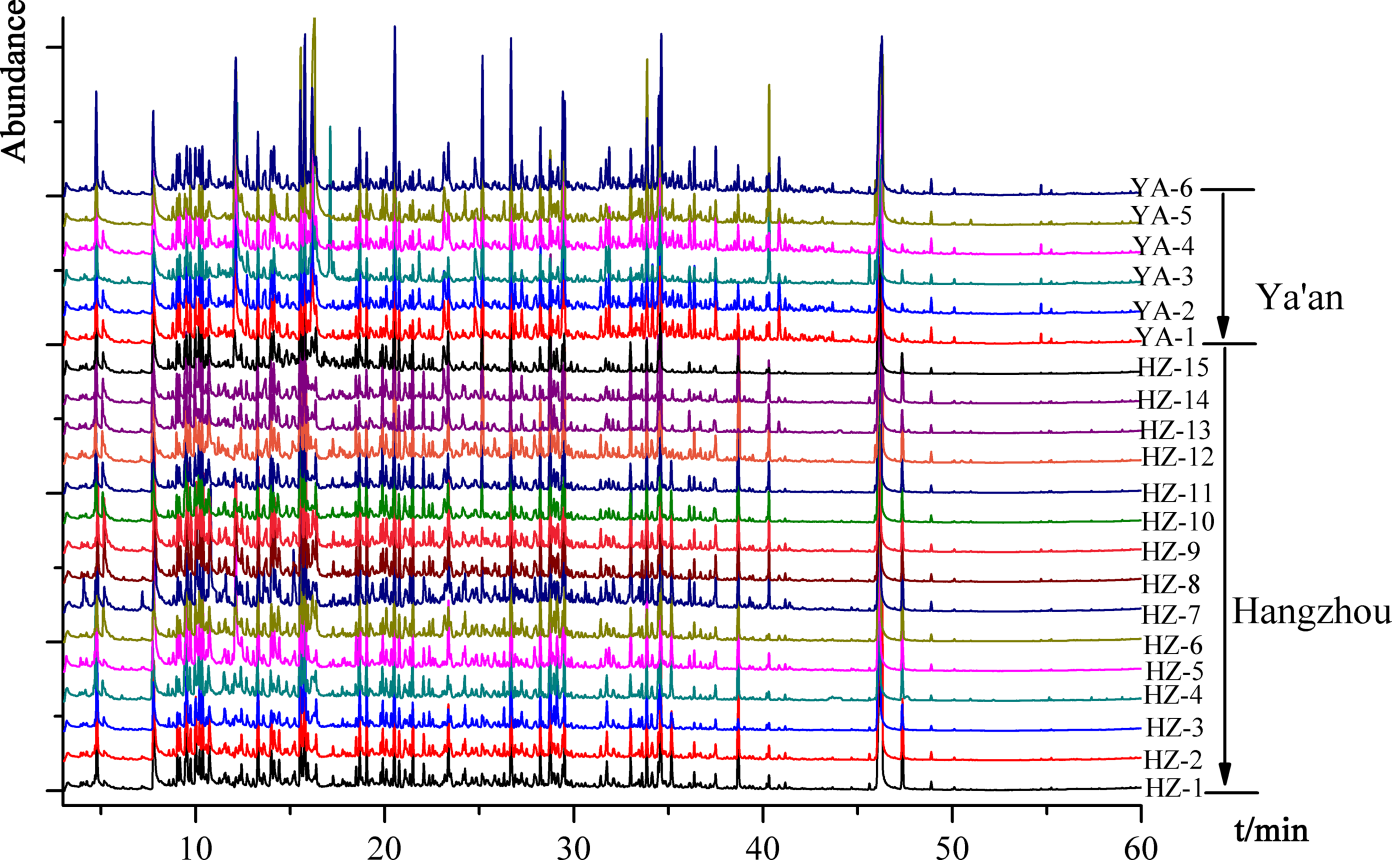
The total ion chromatograms of volatile compounds in green teas from different geographical regions.

On the basis of the optimized conditions, IRAE-HS-SPME was applied to identify the volatile profile of green tea samples from Hangzhou and Ya’an district. The volatile components were identified by comparing the obtained mass spectra with standard ones from NIST Mass Spectral library and by the Kovàts retention indices calculated for each peak with reference to the normal alkanes C7-C40 series. The relative amounts were calculated by the individual peak area relative to the total areas. The GC-MS chromatograms of green teas from different geographical origins were mapped in Fig 3. A total of 82 volatile compounds were identified in 21 green tea samples, including 27 hydrocarbons, 2 furans, 9 alcohols, 11 ketones, 7 esters, 17 aldehydes, 9 nitrogen compounds (see Table 3). As seen from S1 Fig, significant differences were observed in terms of relative area percentages of the different categories of volatile compounds between Hangzhou and Ya’an district.

**Table 3:** Volatile compounds and their relative contents in green tea from different geographical regions.

					Relative percentage content [% (range)]			
No.	RI^a^	RI^b^	Compound^c^	ID^d^	Hangzhou (n = 15)	Ya’an (n = 6)	*p* value^e^	VIP
**1**	859	861	1-Hexanol	MS,RI	0.26±0.31	0.69±0.40	0.016	1.29
**2**	878		1-(1-Cyclohexen-1-yl)ethanone*	MS	0.12±0.04	0.06±0.02	**0.002**	**1.59**
**3**	881	884	2-Heptanone	MS,RI	0.13±0.04	0.09±0.03	0.040	1.17
**4**	902	903	Heptanal	MS,RI	3.67±1.24	4.74±2.04	0.154	0.84
**5**	909	905	2,5-Dimethylpyrazine*	MS,RI	1.44±0.77	0.31±0.42	**0.003**	**1.52**
**6**	913	907	Ethylpyrazine	MS,RI	0.10±0.15	0.01±0.03	0.161	0.80
**7**	957	951	(E)-2-Heptenal	MS,RI	3.74±0.81	2.69±0.85	0.016	1.35
**8**	959	957	Benzaldehyde	MS,RI	2.66±0.60	3.51±0.85	0.018	1.28
**9**	963	968	5-Methyl-2-furaldehyde	MS,RI	0.15±0.09	0.12±0.04	0.427	0.58
**10**	971		6,6-Dimethyl-undecane	MS	0.11±0.14	0.08±0.10	0.617	0.41
**11**	975		3,5,5-Trimethyl-2-hexene	MS	0.38±0.11	0.48±0.10	0.089	1.11
**12**	979		1-Hepten-3-one	MS	1.21±0.31	1.00±0.28	0.178	0.91
**13**	982	979	1-Octen-3-ol	MS,RI	1.55±0.87	1.37±0.23	0.640	0.31
**14**	992	989	2-Pentyl-furan	MS,RI	1.19±0.42	1.01±0.25	0.354	0.54
**15**	997	990	(E,E)-2,4-Heptadienal*	MS,RI	2.18±0.52	1.25±0.64	**0.003**	**1.57**
**16**	998	993	2-Ethyl-5-methylpyrazine	MS,RI	0.49±0.43	0.40±0.07	0.430	0.29
**17**	1000	1000	Decane	MS,RI	1.66±0.70	1.10±0.68	0.117	0.89
**18**	1001	1006	Octanal	MS,RI	2.05±0.58	1.54±0.37	0.058	1.08
**19**	1004	1012	α-Terpinene*	MS,RI	1.85±0.39	1.16±0.42	**0.002**	**1.59**
**20**	1023	1016	2-(2-Propenyl)-furan	MS,RI	0.42±0.43	0.26±0.16	0.399	0.51
**21**	1026	1026	D-Limonene	MS,RI	0.44±0.16	0.34±0.15	0.215	0.76
**22**	1035	1034	Benzyl alcohol*	MS,RI	3.02±2.69	12.84±3.84	**0.000**	**2.09**
**23**	1036		(E)-4-Oxohex-2-enal	MS	0.14±0.21	0.00±0.00	0.129	0.87
**24**	1040		3-Octen-2-one	MS	0.29±0.17	0.34±0.27	0.606	0.32
**25**	1042	1039	Benzeneacetaldehyde	MS,RI	1.13±0.44	1.34±0.83	0.575	0.44
**26**	1047		1-Ethyl-1H-pyrrole-2-carbaldehyde	MS	0.79±0.83	1.03±0.20	0.493	0.42
**27**	1049	1043	β-Ocimene	MS,RI	0.22±0.47	0.62±0.48	0.095	0.93
**28**	1059	1049	(E)-2-Octenal	MS,RI	2.57±1.07	2.10±0.56	0.319	0.60
**29**	1064	1064	Acetophenone	MS,RI	0.16±0.35	0.15±0.24	0.972	0.13
**30**	1070	1068	1-(1H-pyrrol-2-yl)ethanone	MS,RI	0.77±0.44	0.51±0.38	0.221	0.70
**31**	1075	1072	1-Octanol	MS,RI	1.51±0.68	0.85±0.77	0.069	1.01
**32**	1078	1082	3-Ethyl-2,5-dimethylpyrazine	MS,RI	0.56±0.58	0.20±0.32	0.169	0.79
**33**	1085	1086	2,6-Diethylpyrazine	MS,RI	0.15±0.18	0.28±0.34	0.271	0.68
**34**	1093	1092	3,5-Octadien-2-one	MS,RI	0.44±0.39	0.08±0.10	0.046	1.10
**35**	1100	1100	Undecane	MS,RI	7.06±3.80	3.04±2.39	0.027	1.20
**36**	1101	1090	Linalool oxide	MS,RI	0.04±0.09	0.00±0.00	0.349	0.59
**37**	1106	1104	Nonanal	MS,RI	7.30±1.55	6.99±2.19	0.716	0.27
**38**	1111	1110	Phenylethyl Alcohol	MS,RI	0.20±0.24	0.00±0.00	0.006	1.09
**39**	1134	1137	1-Ethyl-2,5-pyrrolidinedione	MS,RI	0.44±0.16	0.52±0.30	0.583	0.45
**40**	1151	1152	(E,E)-2,6-Nonadienal	MS,RI	0.14±0.08	0.17±0.10	0.417	0.47
**41**	1157	1159	(E)-2-Nonenal	MS,RI	0.74±0.22	0.58±0.11	0.042	0.90
**42**	1167		3-Methyl-undecane	MS	1.31±0.64	0.73±0.33	0.051	1.07
**43**	1184	1179	(Z)-3-Hexenyl butanoate	MS,RI	0.94±0.54	0.34±0.35	0.023	1.23
**44**	1185	1188	L-α-Terpineol	MS,RI	0.03±0.11	0.09±0.16	0.300	0.62
**45**	1187	1190	Methyl salicylate	MS,RI	0.37±0.13	0.42±0.22	0.558	0.40
**46**	1200	1200	Dodecane	MS,RI	4.25±1.64	4.75±3.32	0.644	0.37
**47**	1201	1205	Decanal	MS,RI	1.11±0.15	0.91±0.15	0.013	1.33
**48**	1214	1218	β-Cyclocitral*	MS,RI	0.53±0.12	0.33±0.13	**0.002**	**1.57**
**49**	1220		1-Phenyl-2-butanone	MS	0.07±0.12	0.00±0.00	0.037	0.81
**50**	1230		n-Valeric acid cis-3-hexenyl ester*	MS	0.75±0.31	0.14±0.13	**0.000**	**1.82**
**51**	1253	1256	Geraniol	MS,RI	0.61±0.82	0.30±0.74	0.441	0.46
**52**	1258	1263	(E)-2-Decenal	MS,RI	1.84±0.54	1.29±0.61	0.056	1.06
**53**	1289	1290	Indole*	MS,RI	0.14±0.19	0.86±0.73	**0.002**	**1.59**
**54**	1300	1300	Tridecane	MS,RI	0.53±1.14	1.01±1.69	0.457	0.43
**55**	1301		2-Methyl-naphthalene	MS	0.12±0.13	0.08±0.02	0.496	0.41
**56**	1334		7-Methyl-heptadecane	MS	0.14±0.20	0.19±0.23	0.621	0.29
**57**	1346	1334	α-Cubebene	MS,RI	0.50±0.18	0.57±0.31	0.643	0.35
**58**	1347	1348	1, 1, 5-Trimethyl-1,2- dihydronaphthalene	MS,RI	0.22±0.16	0.17±0.06	0.468	0.44
**59**	1352	1351	α-Ionene	MS,RI	0.18±0.10	0.14±0.10	0.435	0.46
**60**	1353		5-Methyl-tridecane	MS	0.32±0.43	0.21±0.10	0.525	0.39
**61**	1362		2-Undecenal	MS	0.54±0.23	0.39±0.16	0.145	0.87
**62**	1369		4-Methyl-tetradecane	MS	1.46±0.65	1.04±0.57	0.178	0.78
**63**	1382	1383	(Z)-3-Hexenyl hexanoate*	MS,RI	5.11±1.37	1.04±0.67	**0.000**	**2.11**
**64**	1391	1391	1-Tetradecanol	MS,RI	0.47±1.13	0.41±0.32	0.890	0.20
**65**	1397	1397	Cis-jasmone*	MS,RI	0.98±0.49	3.08±0.97	**0.000**	**2.09**
**66**	1400	1400	Tetradecane	MS,RI	1.97±0.77	2.27±0.72	0.428	0.48
**67**	1426	1428	α-Ionone	MS,RI	0.15±0.42	0.02±0.06	0.49	0.45
**68**	1433	1435	Coumarin	MS,RI	0.41±1.41	0.03±0.05	0.525	0.40
**69**	1453	1452	Geranyl acetone	MS,RI	0.30±0.25	0.67±0.24	0.006	1.45
**70**	1462		2,6,10-Trimethyltridecane	MS	0.55±0.32	0.46±0.20	0.532	0.37
**71**	1485	1487	β-Ionone	MS,RI	1.01±0.37	0.90±0.49	0.569	0.39
**72**	1500	1500	Pentadecane	MS,RI	0.39±0.12	0.57±0.17	0.011	1.36
**73**	1522	1522	Calamenene	MS,RI	0.91±0.35	1.56±0.57	0.005	1.47
**74**	1523	1523	δ-Cadinene*	MS,RI	0.73±0.30	1.48±0.46	**0.000**	**1.77**
**75**	1540	1541	α-Calacorene	MS,RI	0.03±0.09	0.15±0.17	0.150	1.08
**76**	1571		3-Methylpentadecane	MS	0.64±0.82	0.72±0.34	0.819	0.21
**77**	1578		(Z)-3-hexenyl octanoate	MS	0.33±0.44	0.36±0.57	0.897	0.29
**78**	1600	1600	Hexadecane*	MS,RI	0.52±0.17	1.04±0.41	**0.000**	**1.75**
**79**	1672	1673	Cadalene	MS,RI	0.19±0.12	0.34±0.10	0.021	1.25
**80**	1705	1702	(E)-Stilbene	MS,RI	0.01±0.01	0.02±0.01	0.117	0.88
**81**	1846	1840	Caffeine	MS,RI	14.54±4.90	15.44±3.30	0.685	0.33
**82**	1926	1927	Hexadecanoic acid, methyl ester	MS,RI	0.21±0.11	0.29±0.13	0.161	0.80
**Alcohols**					7.68	16.55		
**Nitrogen compounds**					18.64	18.53		
**Furans**					1.61	1.27		
**Aldehydes**					31.29	28.98		
**Hydrocarbons**					26.81	24.07		
**Ketones**					4.85	6.41		
**Esters**					8.11	2.61		
**Others**					1.00	1.57		

^a^RI, retention indices as determined on HP-5MS column using the homologous series of n-alkanes (C7−C40).

^b^RI, retention indices found in literature

^c^Compounds are listed in order of retention time.

^d^Method of identification: MS, identified by comparison with mass spectra; RI, identified by retention indices.

^e^The *p* value by one-way ANOVA.

* Potential markers are marked in bold type letter. It accomplishes: highest significant value (*p*< 0.01) in one-way ANOVA and Variable Important Projection (VIP) >1.50.

A total of 14 saturated and 13 unsaturated hydrocarbons were identified in 21 batches of green teas, accounting for 26.81% and 24.07% in Hangzhou and Ya’an district, respectively. There were significant differences (P<0.05) in the content of hydrocarbon compounds, such as α-terpinene, undecane, pentadecane, calamenene, δ-cadinene, hexadecane, and cadalene. Saturated hydrocarbons are considered to have little contribution to tea flavor, while, unsaturated hydrocarbons play an important role in the flavor of tea [9]. For example, α-terpinene, which generally has a sweet and flowery aroma, is important for green tea’s quality.

The results indicated that aldehydes were present with high proportion in green tea, representing 31.29% and 28.98% in Hangzhou and Ya’an district, respectively. Significant differences (P<0.05) in the content from the two regions were observed, including (E)-2-heptenal, benzaldehyde, (E,E)-2,4-heptadienal, decanal, β-cyclocitral, and (E)-2-nonenal. The aldehydes, originated from thermal Strecker oxidative degradation of amino acids and fatty acids, play an important role on the entire odor because of their relatively low odor threshold values [31]. Typically, benzaldehyde is described as fragrant, sweet, and almond aroma while benzeneacetaldehyde is described as honey-like and sweet.

Among the 11 identified ketones, 1-hepten-3-one, cis-jasmone, β-ionone, were detected as the major ketones, providing a special floral and woody odor. In particular, β-ionone, which can be produced either by enzymatic reactions during fermentation or by thermal degradation, is a significant contributor to the flavor of green tea due to low human odor perception thresholds [32]. Other compounds show significantly difference (P<0.05) including 1-(1-cyclohexen-1-yl)ethanone, 2-heptanone and geranyl acetone.

As far as alcohols are concerned, 1-octen-3-ol, benzyl alcohol, 1-octanol are the most abundant. These results are different from the previous reports [32], which can be attributed to the difference of the extraction method and sample used in this analysis. Benzyl alcohol, which imparts a mild sweet and roasted odor, has been reported in various types of tea. There is no significant difference in the content with regard to α-terpineol, 1-octen-3-ol, and linalool oxide between Hangzhou and Ya’an district. α-Terpineol is a very important odorant that provide a floral and sweet scent. 1-Octen-3-ol, which stems from the oxidative degradation of linoleic acid, is described with an intense, persistent mushroom-like, and earthy odor [31].

A total of 9 nitrogen compounds were identified. Caffeine, which is related to the taste of tea but contributed little to the aroma of tea, is most abundant for all green teas, comprising14.54% and 15.44% for Hangzhou and Ya’an district, respectively. Indole with typical flowery fragrance, is assumed to contribute the overall green tea odor. Pyrazines, such as 2,5-dimethylpyrazine, 3-ethyl-2,5-dimethylpyrazine, which are reported to be formed by Maillard reaction through Strecker degradation, contribute desirable roasty, sweet, and nutty odors [32].

As for the rest of the identified compounds, methyl salicylate, which has a holly oil herbal fragrance, was found to be an important aroma compound. In addition, 2-pentyl-furan and 2-(2-propenyl)-furan with a burnt and sweet odor were identified in the present study [33]. No significant differences (P>0.05) were found in the content for the two furans between Hangzhou and Ya’an district.

### Analysis of the volatile components in green tea and comparison with conventional methods

To evaluate the extraction efficiency, IRAE was compared with conventional water-bath heating method. There are 53 components identified in green tea by water-bath heating (see S2 Table). The chemical classes obtained by water-bath heating were mainly made up of aldehydes (32.04%), hydrocarbons (27.43%) and nitrogenous compounds (14.05%), with relatively small amounts of esters (11.77%), alcohols (5.09%), ketones (5.09%) and furans (3.59%).

**Fig 4:**
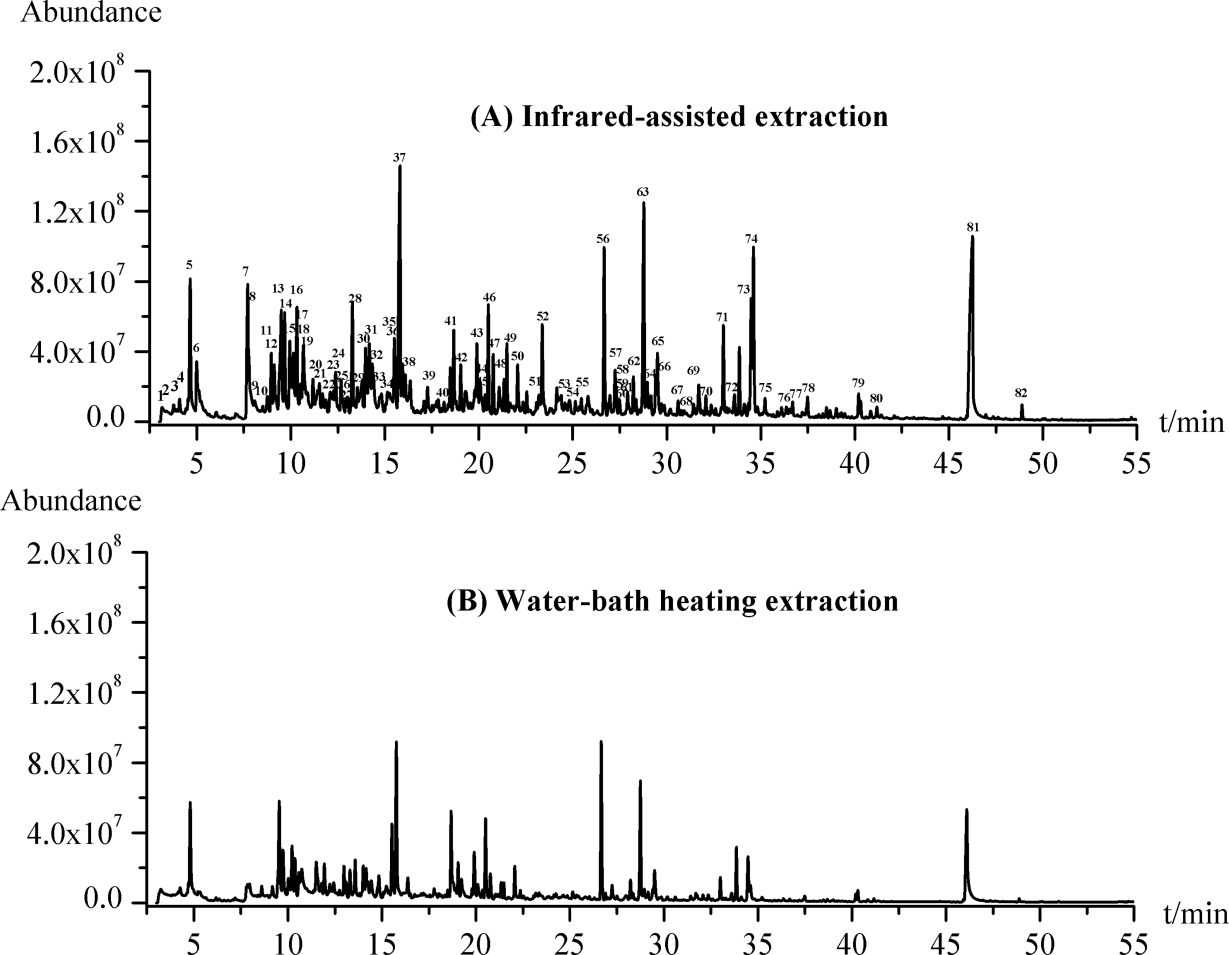
The total ion chromatograms of volatile compounds in green tea obtained by different methods.

It is noteworthy that the IRAE contains most of the volatile components identified by water-bath heating. Moreover, the IRAE-HS-SPME has much higher extraction efficiencies than water-bath heating. Under the conditions investigated, the total peak area obtained by IRAE-HS-SPME is 15.01 ×10^9^ while the total peak area obtained by water-bath heating is 6.23×10^9^ (see Fig 4). The result indicates that the IRAE is superior to the classical water-bath heating method due to the special mechanism of IRAE. The IR radiation emits a continuous spectrum, especially the wavelength range of 2.5–25μm (corresponding wave number range of 400–4000 cm^-1^) which can excite the vibrations in molecules in the modes of stretching, bending, rocking and twisting [34]. The presence of C-H and O-H bonds (such as hydrocarbons and alcohols in green tea) leads to a very strong absorption of infrared radiation between 3100 and 3600 cm^-1^. Additionally, most absorption peaks of volatile components also fall into the range of the IR radiation. That is to say, the wavelength of the infrared radiation matches the absorption characteristics of the active compounds in green tea. In addition, IR radiation owns high penetration capability, it heats the green tea sample in a three-dimension manner without heating the surrounding air, while a finite period of time is needed to heat the vessel before the heat is transferred to the sample in conventional heating mode [35,36]. Therefore, the IRAE provides an alternative, powerful and effective method for the extraction of the volatile in green tea.

### Method precision

The repeatability of the IRAE-HS-SPME method was determined by performing six replicate analyses on HZ-10 green tea sample under the optimum conditions. Ten frequent volatile components in green tea were selected. For these components, the relative standard deviations (RSDs) of retention time and peak area in green tea sample were calculated. As indicated in S3 Table, the RSDs of the retention times of each compound are less than 1%, and the RSDs of the peak area of each compound are less than 8%. These satisfying RSD values definitely prove the stability, indicating that the developed IRAE-HS-SPME method is a reliable method for the determination of the volatile components in green tea samples.

Above all, the IRAE-HS-SPME method is a rapid, high-efficient, solvent-free method for analysis of the volatile components in green tea. It is therefore considered that this method is promising and can be a good alternative to the traditional techniques.

### Multivariate analysis

Multivariate analysis was implemented on the whole data consisting of a 21×82 matrix. The columns represent the green tea samples analysed and the rows represent the relative contents of the volatile metabolites determined by IRAE-HS-SPME. In particular, two classification models were built, in order to characterize the differences related to the geographical origins of the samples.

#### Partial least squares-discrimination analysis

**Fig 5:**
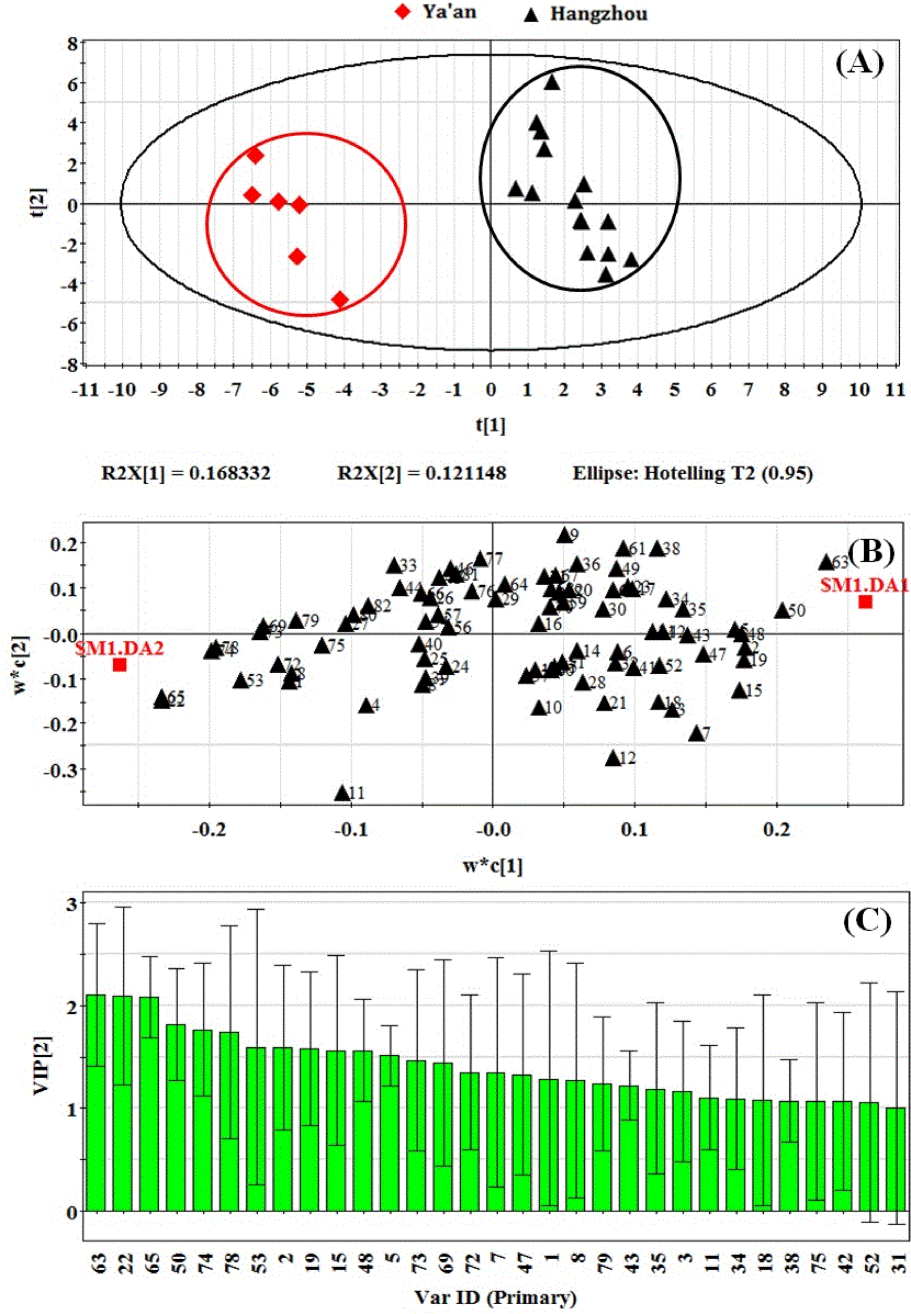
(A) The PLS-DA score plot of component 1 and 2, R2X[1] = 0.168 and R2X[2] = 0.121. (B) The corresponding PLS-DA loading plot of component 1 and 2. The number along with the triangles referred to compound number in Table 3. $M1.DA1 and 2 represent Hangzhou and Ya’an, respectively. (C) The variables important in the projection (VIP) scores, the number referred to compound number in Table 3.

PLS-DA is a multivariate technique used to classify different groups of samples. It is based on linking two data matrices, X (explanatory dataset) and Y (explicative dataset). In this study, two types of samples (a total of twenty-one) were processed using PLS-DA method. As shown in Fig 5A, a significant discrimination between Hangzhou and Ya’an of green teas according to the data matrix of their volatile compounds was observed by using a PLS-DA model, one group for the sample dots of Hangzhou (positive position) and another one for the sample dots of Ya’an (negative position).The well-explained variance (R2Y = 0.978) and cross-validated predictive capability (Q2 = 0.838) indicates the model’s good feasibility.

PLS-DA was further performed to display specific volatile components that better explain the differences of the two regions. The results of PLS-DA highly coincide with the comparison result of the concentration. For example, some volatile compounds such as (Z)-3-hexenyl hexanoate (63), n-valeric acid cis-3-hexenyl ester (50), 2,5-dimethylpyrazine (5), α-terpinene (19), (E,E)-2,4-heptadienal (15) and (Z)-3-hexenyl butanoate (43) presenting higher concentrations in Hangzhou samples than in Ya’an samples (see Table 3) are strongly correlated with Hangzhou green teas and located in the positive position of w*c axis (Fig 5B). Similarly, several specific compounds located in the negative position of w*c axis such as cis-jasmone (65), benzyl alcohol (22), calamenene (73), indole (53), and hexadecane (78) may contribute to the specificity of Ya’an samples and they also display higher contents in Ya’an green teas when compared with that in Hangzhou green teas.

Subsequently, the variable influence on the projection (VIP) parameter was used to select the metabolites which exhibited significant contribution in discriminating the two groups in PLS-DA model. Indeed, the VIP score is an index accounting for the relative importance: the higher the value of the VIP score, the more relevant the variable. Moreover, the VIP scores are normalized in a way that their average value on a particular model is always 1, so that a “larger than 1” criterion can be adopted to assess the significance of the contributions of individual predictors [37]. In the present study, a total of 31 differential compounds with VIP value larger than 1 were screened out (see Fig 5C), indicating that they have above average influence on the differentiating green teas from Hangzhou and Ya’an regions.

#### Hierarchical clustering analysis

**Fig 6:**
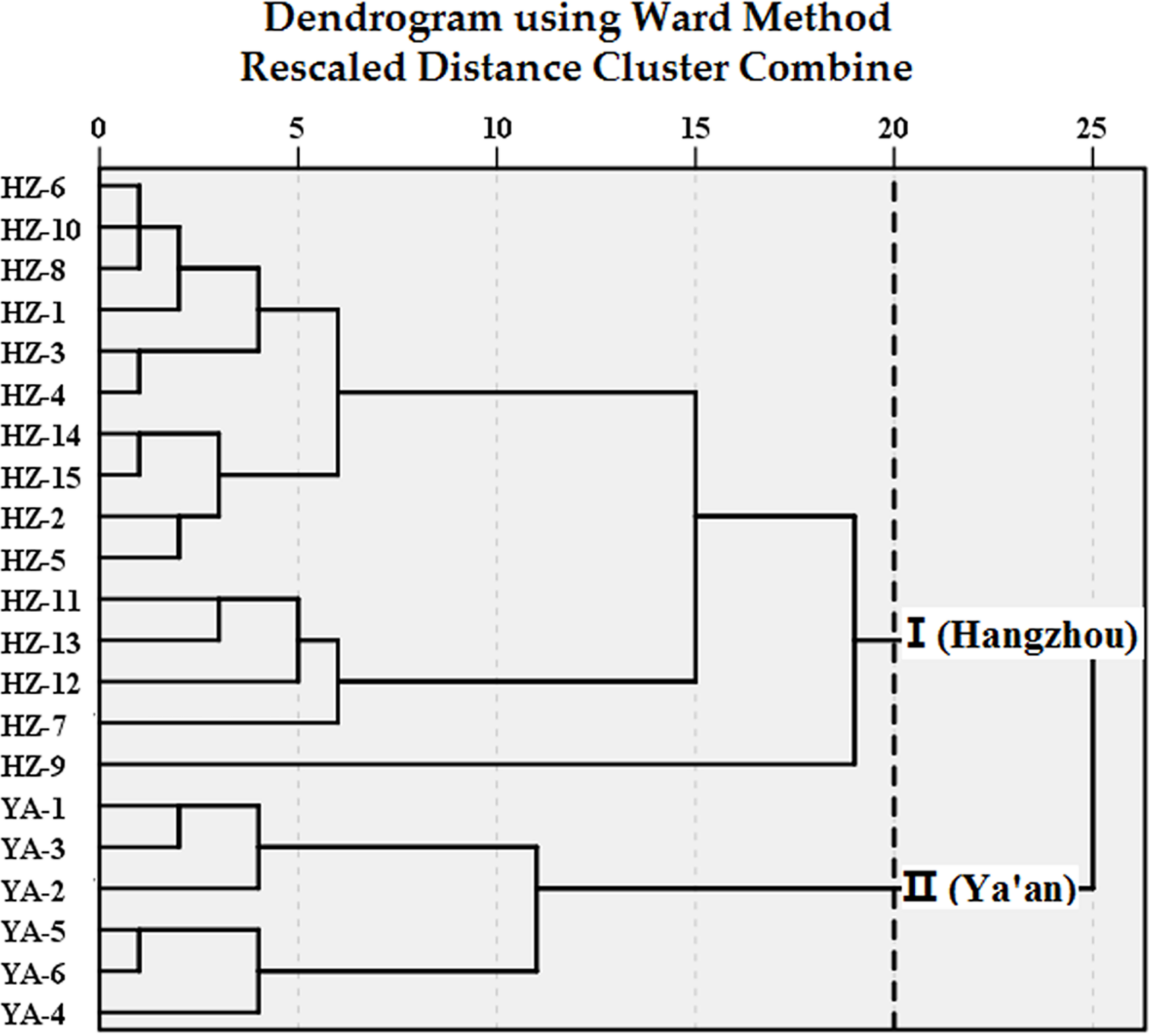
Cluster dendrogram of tea samples from different regions.

HCA is an ideal technique for the crude classification of tea samples based on the contents of volatile components because it does not require previous information of test samples [38]. HCA of 21 samples was performed using a Ward’s method to visualize the differences and/or similarities among samples through Squared Euclidean distance. At a distance level of 20, all the samples can be clustered into two groups (see Fig 6). The first cluster I consists of 15 samples (HZ-1 to HZ-15) from Hangzhou district. The second cluster II is made up of six samples (YA-1 to YA-6) from Ya’an district. It is probably because of some conceivable reasons, such as the different cultivation region, soil, and climatic conditions, harvesting seasons, processing methods, and other factors. The displaying procedures of HCA produced consistent result with that of PLS-DA, providing enough information to discriminate green tea samples from different regions.

### Potential volatile markers

The application of a dual criterion, based on the univariate and multivariate analysis, allows the identification of potential volatile markers for different regions [39]. In our model, the potential volatile markers (see Table 3) were identified based on the variable importance in the projection values (VIP > 1.50) and on the category from one-way ANOVA (significant level P<0.01). According to this, 12 volatile compounds including (Z)-3-hexenyl hexanoate (63), benzyl alcohol (22), cis-jasmone (65), n-valeric acid cis-3-hexenyl ester (50), δ-cadinene (74), hexadecane (78), indole (53), 1-(1-cyclohexen-1-yl)ethanone (2), α-terpinene (19), (E,E)-2,4-heptadienal (15), β-cyclocitral (48) and 2,5-dimethylpyrazine (5) are the main compounds for discriminating between the cultivation systems. Among them, benzyl alcohol, α-terpinene and (E,E)-2,4-heptadienal plays an important role in the flavor of green tea. We should remark that our study is based on two widely known cultivars. Further studies should be carried out with a higher number of cultivars to correlate the same behaviour in different systems.

## Conclusions

In the present work, a novel IRAE-HS-SPME followed by GC-MS was developed for rapid determination of the volatile components in green teas. The optimal IRAE-HS-SPME performance was obtained under operating conditions: CAR-PDMS fiber, sample amount 0.5 g, extraction time 20 min, IR power 175 W and IR lamp distance of 6 cm. A total of 82 volatile components were extracted and identified in 21 green tea samples by the proposed technique. Multivariate technique including PLS-DA and HCA were successfully employed to provide a visual comparison and highlight the differences in the volatiles profiling of green teas from different geographical origins. Furthermore, 12 potential volatile markers were identified to be the most important variables in distinguishing the samples based on the variable importance in the projection values (VIP > 1.50) and on the category from one-way ANOVA (P<0.01). The results shows that the combination of IRAE-HS-SPME/GC-MS technique to multivariate analysis offers a valuable tool to assess geographical traceability of different green tea samples.

## Supporting information

S1 Fig The relative contents of compounds type in green teas from different geographical regions. (TIF)

S1 Table The details of 21 green tea samples in the experiment. (DOC)

S2 Table The volatile components in green tea obtained by water-bath heating extraction. (DOC)

S3 Table The RSD values of volatile components in HZ-10 green tea sample (n = 6). (DOC)
