# Supplementary material for: Characterization of the volatile components in green tea by IRAE-HS-SPME/GC-MS combined with multivariate analysis
Source: PLoS One. 2018 Mar 1;13(3):e0193393. doi: 10.1371/journal.pone.0193393 (PMC5832268; doi:10.1371/journal.pone.0193393)
Supplement: S1 Table — (DOC) [file pone.0193393.s002.doc]

***Supporting Information for***

**Characterization of the Volatile Components in Green Tea by IRAE-HS-SPME/GC-MS Combined with Multivariate Analysis**

**Yan-Qin Yang^1^, Hong-Xu Yin^1^, Hai-Bo Yuan****^1,*^, Yong-Wen Jiang^1,*^,**

**Chun-Wang Dong^1^, Yu-Liang Deng^1^**

^1^Key Laboratory of Tea Biology and Resources Utilization, Ministry of Agriculture, Tea Research Institute, Chinese Academy of Agricultural Sciences, Hangzhou, Zhejiang China

*****Corresponding Author:

E-Mail: [jiangyw@tricaas.com](mailto:jiangyw@tricaas.com) (YWJ), 192168092@ tricaas.com (HBY)

**S1 Table. The details of 21** green tea samples in the experiment

| **No.** | **Name** | **Regions** | **Grades** | **Harvest time** |
| --- | --- | --- | --- | --- |
| HZ-1 | Longing tea | Hangzhou district, Zhejiang Province | Grade Super | 2016 |
| HZ -2 | Longing tea | Hangzhou district, Zhejiang Province | Grade One | 2016 |
| HZ -3 | Longing tea | Hangzhou district, Zhejiang Province | Grade One | 2016 |
| HZ -4 | Longing tea | Hangzhou district, Zhejiang Province | Grade One | 2016 |
| HZ -5 | Longing tea | Hangzhou district, Zhejiang Province | Grade One | 2016 |
| HZ -6 | Longing tea | Hangzhou district, Zhejiang Province | Grade One | 2016 |
| HZ -7 | Longing tea | Hangzhou district, Zhejiang Province | Grade One | 2016 |
| HZ -8 | Longing tea | Hangzhou district, Zhejiang Province | Grade Super | 2016 |
| HZ -9 | Longing tea | Hangzhou district, Zhejiang Province | Grade Super | 2016 |
| HZ -10 | Longing tea | Hangzhou district, Zhejiang Province | Grade Super | 2016 |
| HZ -11 | Longing tea | Hangzhou district, Zhejiang Province | Grade Super | 2016 |
| HZ -12 | Longing tea | Hangzhou district, Zhejiang Province | Grade Super | 2016 |
| HZ -13 | Longing tea | Hangzhou district, Zhejiang Province | Grade Super | 2016 |
| HZ -14 | Longing tea | Hangzhou district, Zhejiang Province | Grade Super | 2016 |
| HZ -15 | Longing tea | Hangzhou district, Zhejiang Province | Grade Super | 2016 |
| YA-1 | Mengding tea | Ya’an district, Sichuan Province | Grade Super | 2016 |
| YA -2 | Mengding tea | Ya’an district, Sichuan Province | Grade Super | 2016 |
| YA -3 | Mengding tea | Ya’an district, Sichuan Province | Grade Super | 2016 |
| YA -4 | Mengding tea | Ya’an district, Sichuan Province | Grade Super | 2016 |
| YA -5 | Mengding tea | Ya’an district, Sichuan Province | Grade Super | 2016 |
| YA -6 | Mengding tea | Ya’an district, Sichuan Province | Grade Super | 2016 |
