# Supplementary material for: Characterization of the volatile components in green tea by IRAE-HS-SPME/GC-MS combined with multivariate analysis
Source: PLoS One. 2018 Mar 1;13(3):e0193393. doi: 10.1371/journal.pone.0193393 (PMC5832268; doi:10.1371/journal.pone.0193393)
Supplement: S2 Table — (DOC) [file pone.0193393.s003.doc]

***Supporting Information for***

**Characterization of the Volatile Components in Green Tea by IRAE-HS-SPME/GC-MS Combined with Multivariate Analysis**

**Yan-Qin Yang^1^, Hong-Xu Yin^1^, Hai-Bo Yuan****^1,*^, Yong-Wen Jiang^1,*^,**

**Chun-Wang Dong^1^, Yu-Liang Deng^1^**

^1^Key Laboratory of Tea Biology and Resources Utilization, Ministry of Agriculture, Tea Research Institute, Chinese Academy of Agricultural Sciences, Hangzhou, Zhejiang China

*****Corresponding Author:

E-Mail: [jiangyw@tricaas.com](mailto:jiangyw@tricaas.com) (YWJ), 192168092@ tricaas.com (HBY)

**S2 Table. The volatile components in green tea obtained by water-bath heating extraction**

| **No.** | **RI^a^** | **RI^b^** | **Compound^c^** | **ID^d^** | **Relative percentage content [% (range)]** |
| --- | --- | --- | --- | --- | --- |
| 1 | 859 | 861 | 1-Hexanol | MS,RI | 0.89 |
| 2 | 878 |  | 1-(1-Cyclohexen-1-yl)ethanone | MS | -- |
| 3 | 881 | 884 | 2-Heptanone | MS,RI | 0.29 |
| 4 | 902 | 903 | Heptanal | MS,RI | 7.63 |
| 5 | 909 | 905 | 2,5-Dimethylpyrazine | MS,RI | 0.77 |
| 6 | 913 | 907 | Ethylpyrazine | MS,RI | -- |
| 7 | 957 | 951 | (E)-2-Heptenal | MS,RI | -- |
| 8 | 959 | 957 | Benzaldehyde | MS,RI | 3.17 |
| 9 | 963 | 968 | 5-Methyl-2-furaldehyde | MS,RI | -- |
| 10 | 971 |  | 6,6-Dimethyl-undecane | MS | 0.80 |
| 11 | 975 |  | 3,5,5-Trimethyl-2-hexene | MS | -- |
| 12 | 979 |  | 1-Hepten-3-one | MS | -- |
| 13 | 982 | 979 | 1-Octen-3-ol | MS,RI | 1.09 |
| 14 | 992 | 989 | 2-Pentyl-furan | MS,RI | 3.48 |
| 15 | 997 | 990 | (E,E)-2,4-Heptadienal | MS,RI | 1.58 |
| 16 | 998 | 993 | 2-Ethyl-5-methylpyrazine | MS,RI | 0.25 |
| 17 | 1000 | 1000 | Decane | MS,RI | 3.38 |
| 18 | 1001 | 1006 | Octanal | MS,RI | 2.91 |
| 19 | 1004 | 1012 | α-Terpinene | MS,RI | 1.84 |
| 20 | 1023 | 1016 | 2-(2-Propenyl)-furan | MS,RI | 0.11 |
| 21 | 1026 | 1026 | D-Limonene | MS,RI | 0.86 |
| 22 | 1035 | 1034 | Benzyl alcohol | MS,RI | -- |
| 23 | 1036 |  | (E)-4-Oxohex-2-enal | MS | -- |
| 24 | 1040 |  | 3-Octen-2-one | MS | 0.37 |
| 25 | 1042 | 1039 | Benzeneacetaldehyde | MS,RI | 1.45 |
| 26 | 1047 |  | 1-Ethyl-1H-pyrrole-2-carbaldehyde | MS | -- |
| 27 | 1049 | 1043 | β-Ocimene | MS,RI | -- |
| 28 | 1059 | 1049 | (E)-2-Octenal | MS,RI | -- |
| 29 | 1064 | 1064 | Acetophenone | MS,RI | -- |
| 30 | 1070 | 1068 | 1-(1H-pyrrol-2-yl)ethanone | MS,RI | -- |
| 31 | 1075 | 1072 | 1-Octanol | MS,RI | 0.69 |
| 32 | 1078 | 1082 | 3-Ethyl-2,5-dimethylpyrazine | MS,RI | 0.69 |
| 33 | 1085 | 1086 | 2,6-Diethylpyrazine | MS,RI | -- |
| 34 | 1093 | 1092 | 3,5-Octadien-2-one | MS,RI | 2.67 |
| 35 | 1100 | 1100 | Undecane | MS,RI | 4.06 |
| 36 | 1101 | 1090 | Linalool oxide | MS,RI | 1.05 |
| 37 | 1106 | 1104 | Nonanal | MS,RI | 11.86 |
| 38 | 1111 | 1110 | Phenylethyl Alcohol | MS,RI | 0.14 |
| 39 | 1134 | 1137 | 1-Ethyl-2,5-pyrrolidinedione | MS,RI | 0.15 |
| 40 | 1151 | 1152 | (E,E)-2,6-Nonadienal | MS,RI | -- |
| 41 | 1157 | 1159 | (E)-2-Nonenal | MS,RI | 0.49 |
| 42 | 1167 |  | 3-Methyl-undecane | MS | 0.18 |
| 43 | 1184 | 1179 | (Z)-3-Hexenyl butanoate | MS,RI | 2.74 |
| 44 | 1185 | 1188 | L-α-Terpineol | MS,RI | 1.03 |
| 45 | 1187 | 1190 | Methyl salicylate | MS,RI | 0.41 |
| 46 | 1200 | 1200 | Dodecane | MS,RI | 6.22 |
| 47 | 1201 | 1205 | Decanal | MS,RI | 1.91 |
| 48 | 1214 | 1218 | β-Cyclocitral | MS,RI | 1.05 |
| 49 | 1220 |  | 1-Phenyl-2-butanone | MS | -- |
| 50 | 1230 |  | n-Valeric acid cis-3-hexenyl ester | MS | -- |
| 51 | 1253 | 1256 | Geraniol | MS,RI | 0.21 |
| 52 | 1258 | 1263 | (E)-2-Decenal | MS,RI | -- |
| 53 | 1289 | 1290 | Indole | MS,RI | -- |
| 54 | 1300 | 1300 | Tridecane | MS,RI | 0.59 |
| 55 | 1301 |  | 2-Methyl-Naphthalene | MS | 0.12 |
| 56 | 1334 |  | 7-Methyl-heptadecane | MS | 0.34 |
| 57 | 1346 | 1334 | α-Cubebene | MS,RI | 0.78 |
| 58 | 1347 | 1348 | 1, 1, 5-Trimethyl-1, 2-dihydronaphthalene | MS,RI | -- |
| 59 | 1352 | 1351 | α-Ionene | MS,RI | 0.12 |
| 60 | 1353 |  | 5-Methyl-tridecane | MS | 0.14 |
| 61 | 1362 |  | 2-Undecenal | MS | -- |
| 62 | 1369 |  | 4-Methyl-tetradecane | MS | 1.32 |
| 63 | 1382 | 1383 | (Z)-3-Hexenyl hexanoate | MS,RI | 8.62 |
| 64 | 1391 | 1391 | 1-Tetradecanol | MS,RI | -- |
| 65 | 1397 | 1397 | Cis-jasmone | MS,RI | 0.62 |
| 66 | 1400 | 1400 | Tetradecane | MS,RI | 1.73 |
| 67 | 1426 | 1428 | α-Ionone | MS,RI | -- |
| 68 | 1433 | 1435 | Coumarin | MS,RI | -- |
| 69 | 1453 | 1452 | Geranyl acetone | MS,RI | -- |
| 70 | 1462 |  | 2,6,10-Trimethyltridecane | MS | 0.30 |
| 71 | 1485 | 1487 | β-Ionone | MS,RI | 1.15 |
| 72 | 1500 | 1500 | Pentadecane | MS,RI | 0.33 |
| 73 | 1522 | 1522 | Calamenene | MS,RI | 2.11 |
| 74 | 1523 | 1523 | [δ-Cadinene](http://www.basechem.org/chemical/55182) | MS,RI | 1.39 |
| 75 | 1540 | 1541 | α-Calacorene | MS,RI | 0.16 |
| 76 | 1571 |  | 3-Methylpentadecane | MS | -- |
| 77 | 1578 |  | (Z)-3-hexenyl octanoate | MS | -- |
| 78 | 1600 | 1600 | Hexadecane | MS,RI | 0.25 |
| 79 | 1672 | 1673 | Cadalene | MS,RI | 0.40 |
| 80 | 1705 | 1702 | (E)-Stilbene | MS,RI | -- |
| 81 | 1846 | 1840 | Caffeine | MS,RI | 12.20 |
| 82 | 1926 |  | Hexadecanoic acid, methyl ester | MS,RI | -- |
| Others | |  |  |  | 0.91 |

^a^RI, retention indices as determined on HP-5MS column using the homologous series of n-alkanes (C7−C40).

^b^RI, retention indices found in literature

^c^Compounds are listed in order of retention time.

^d^Method of identification: MS, identified by comparison with mass spectra; RI, identified by retention indices
