# Supplementary material for: Characterization of the volatile components in green tea by IRAE-HS-SPME/GC-MS combined with multivariate analysis
Source: PLoS One. 2018 Mar 1;13(3):e0193393. doi: 10.1371/journal.pone.0193393 (PMC5832268; doi:10.1371/journal.pone.0193393)
Supplement: S3 Table — (DOC) [file pone.0193393.s004.doc]

***Supporting Information for***

**Characterization of the Volatile Components in Green Tea by IRAE-HS-SPME/GC-MS Combined with Multivariate Analysis**

**Yan-Qin Yang^1^, Hong-Xu Yin^1^, Hai-Bo Yuan****^1,*^, Yong-Wen Jiang^1,*^,**

**Chun-Wang Dong^1^, Yu-Liang Deng^1^**

^1^Key Laboratory of Tea Biology and Resources Utilization, Ministry of Agriculture, Tea Research Institute, Chinese Academy of Agricultural Sciences, Hangzhou, Zhejiang China

*****Corresponding Author:

E-Mail: [jiangyw@tricaas.com](mailto:jiangyw@tricaas.com) (YWJ), 192168092@ tricaas.com (HBY)

**S3 Table. The RSD values of volatile components in** **HZ-10 green tea sample (n = 6)**

| **Retention time**  **(min)** | **Compound name** | **RSD^a^** | **RSD^b^** |
| --- | --- | --- | --- |
| 7.915 | Benzaldehyde | 0.79% | 5.46% |
| 9.766 | 2-Pentyl-furan | 0.63% | 4.62% |
| 10.051 | (E,E)-2,4-heptadienal | 0.36% | 2.15% |
| 12.102 | Benzyl alcohol | 0.48% | 4.36% |
| 15.847 | Nonanal | 0.71% | 6.87% |
| 20.093 | Methyl salicylate | 0.57% | 7.36% |
| 24.802 | Indole | 0.74% | 6.91% |
| 28.806 | (Z)-3-Hexenyl hexanoate | 0.52% | 4.25% |
| 33.034 | β-ionone | 0.45% | 3.67% |
| 46.600 | Caffeine | 0.69% | 4.86% |

^a^RSD, RSD value of retention time.

^b^RSD, RSD value of peak area.
